# 3-Acetyl-1-(3-chloro­phen­yl)thio­urea

**DOI:** 10.1107/S1600536812012147

**Published:** 2012-03-24

**Authors:** Durre Shahwar, M. Nawaz Tahir, Muhammad Mansha Chohan, Naeem Ahmad, M. Asam Raza

**Affiliations:** a Department of Chemistry, Government College University, Lahore, Pakistan; b University of Sargodha, Department of Physics, Sargodha, Pakistan; c Department of Chemistry, University of Gujrat, Gujrat, Pakistan

## Abstract

In the title compound, C_9_H_9_ClN_2_OS, the 3-chloro­phenyl and acetyl­thio­urea fragments are oriented at a dihedral angle of 62.68 (5)°. An intra­molecular N—H⋯O hydrogen bond generates an *S*(6) ring motif. Mol­ecules are linked into dimers *via* a cyclic *R*_2_^2^(8) motif of N—H⋯S hydrogen bonds. These dimers are further connected through C—H⋯S inter­actions, completing an *R*_2_^2^(12) motif, into chains along [010].

## Related literature

For related structures, see: Shahwar *et al.* (2012**a* ▶,b* ▶).; For graph-set notation, see: Bernstein *et al.* (1995 ▶).
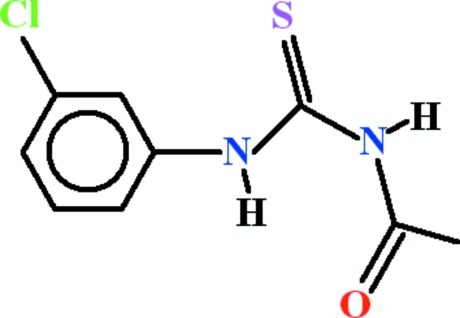


## Experimental

### 

#### Crystal data

C_9_H_9_ClN_2_OS
*M* *_r_* = 228.69
Monoclinic, 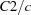

*a* = 28.3980 (14) Å
*b* = 4.1768 (2) Å
*c* = 20.2635 (11) Å
β = 122.651 (2)°
*V* = 2023.69 (18) Å^3^
*Z* = 8
Mo *K*α radiation
μ = 0.55 mm^−1^
*T* = 296 K
0.35 × 0.22 × 0.22 mm

#### Data collection

Bruker Kappa APEXII CCD diffractometer
Absorption correction: multi-scan (*SADABS*; Bruker, 2005 ▶) *T*_min_ = 0.868, *T*_max_ = 0.872
7045 measured reflections
1775 independent reflections
1450 reflections with *I* > 2σ(*I*)
*R*_int_ = 0.029

#### Refinement

*R*[*F* ^2^ > 2σ(*F* ^2^)] = 0.034
*wR*(*F* ^2^) = 0.109
*S* = 1.11
1775 reflections
128 parameters
H-atom parameters constrained
Δρ_max_ = 0.23 e Å^−3^
Δρ_min_ = −0.23 e Å^−3^

### 

Data collection: *APEX2* (Bruker, 2009 ▶); cell refinement: *SAINT* (Bruker, 2009 ▶); data reduction: *SAINT*; program(s) used to solve structure: *SHELXS97* (Sheldrick, 2008 ▶); program(s) used to refine structure: *SHELXL97* (Sheldrick, 2008 ▶); molecular graphics: *ORTEP-3 for Windows* (Farrugia, 1997 ▶) and *PLATON* (Spek, 2009 ▶); software used to prepare material for publication: *WinGX* (Farrugia, 1999 ▶) and *PLATON*.

## Supplementary Material

Crystal structure: contains datablock(s) global, I. DOI: 10.1107/S1600536812012147/gk2470sup1.cif

Structure factors: contains datablock(s) I. DOI: 10.1107/S1600536812012147/gk2470Isup2.hkl

Supplementary material file. DOI: 10.1107/S1600536812012147/gk2470Isup3.cml

Additional supplementary materials:  crystallographic information; 3D view; checkCIF report

## Figures and Tables

**Table 1:** Hydrogen-bond geometry (Å, °)

*D*—H⋯*A*	*D*—H	H⋯*A*	*D*⋯*A*	*D*—H⋯*A*
N1—H1⋯O1	0.86	1.96	2.648 (3)	136
N2—H2⋯S1^i^	0.86	2.56	3.4095 (18)	170
C9—H9*A*⋯S1^ii^	0.96	2.85	3.799 (3)	170

Symmetry codes: (i) 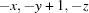; (ii) 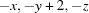.

**Fig. 1.**
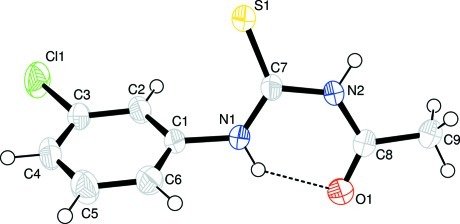
View of the title compound with displacement ellipsoids drawn at the 50% probability level. The dotted lines represent the intramolecular hydrogen bond.

**Fig. 2.**
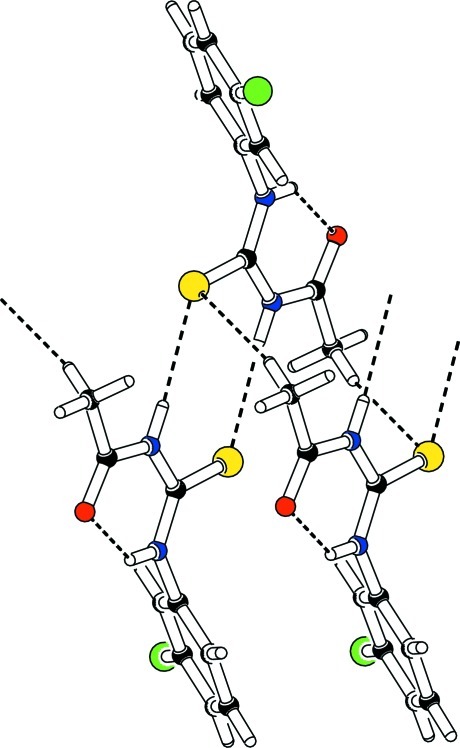
The partial packing (PLATON; Spek, 2009) showing molecules connected via N-H···O and C-H···S interactions.

## supplementary crystallographic information

### Comment

The title compound (Fig. 1) has been synthesized as a continuation of our work to find new enzyme inhibitors.

The crystal structures of *N*-(2-methylphenylcarbamothioyl)acetamide (Shahwar *et al.*, 2012*a*) and *N*-(phenylcarbamothioyl)acetamide (Shahwar *et al.*, 2012*b*) have been reported which are related to the title compound.

In the title compound, the 3-chlorophenyl group A (C1–C6/Cl1) and the *N*-carbamothioylacetamide moiety B (N1/C7/S1/N2/C8/O1/C9) are planar with r. m. s. deviation of 0.0055 Å and 0.0060 Å, respectively. The dihedral angle between A/B is 62.68 (5)°. There exist intramolecular H–bonding of N—H···O type (Table 1, Fig. 1) with *S*(6) ring motif (Bernstein *et al.*, 1995). The molecules are dimerized due to N—H···S type of hydrogen bonds with *R*_2_^2^(8) ring motifs (Table 1, Fig. 2). The dimers are interlinked due to C—H···S H–bondings (Table 1, Fig. 2) and complete *R*_2_^2^(12) ring motifs.

### Experimental

The title compound was synthesized by adding (0.1 mol, 7.13 ml) of acetylchloride dropwise to a stirred solution of KSCN (0.11 mol) in dry acetone (50 ml), followed by slow addition of 3-chloroaniline (0.1 mol) in dry acetone (25 ml). The mixture was refluxed for 5–10 min, then poured on ice cooled water, which resulted in crude precipitate. Recrystallization of the precipitate from ethyl acetate yielded colorless rods (m.p. 374 K).

### Refinement

The H atoms were positioned geometrically (C—H = 0.93–0.96 Å, N—H = 0.86 Å) and refined as riding with *U*_iso_(H) = *xU*_eq_(C, N), where *x* = 1.5 for methyl groups and *x* = 1.2 for other H atoms.

### Crystal data

**Table:**

C_9_H_9_ClN_2_OS	*F*(000) = 944
*M**_r_* = 228.69	*D*_x_ = 1.501 Mg m^−^^3^
Monoclinic, *C*2/*c*	Mo *K*α radiation, λ = 0.71073 Å
Hall symbol: -C 2yc	Cell parameters from 1464 reflections
*a* = 28.3980 (14) Å	θ = 2.1–25.1°
*b* = 4.1768 (2) Å	µ = 0.55 mm^−^^1^
*c* = 20.2635 (11) Å	*T* = 296 K
β = 122.651 (2)°	Rod, colorless
*V* = 2023.69 (18) Å^3^	0.35 × 0.22 × 0.22 mm
*Z* = 8	

### Data collection

**Table:**

Bruker Kappa APEXII CCD diffractometer	1745 independent reflections
Radiation source: fine-focus sealed tube	1450 reflections with *I* > 2σ(*I*)
Graphite monochromator	*R*_int_ = 0.029
Detector resolution: 8.10 pixels mm^-1^	θ_max_ = 25.1°, θ_min_ = 2.1°
ω scans	*h* = −33→27
Absorption correction: multi-scan (*SADABS*; Bruker, 2005)	*k* = −4→3
*T*_min_ = 0.868, *T*_max_ = 0.872	*l* = −19→24
7045 measured reflections	

### Refinement

**Table:**

Refinement on *F*^2^	Primary atom site location: structure-invariant direct methods
Least-squares matrix: full	Secondary atom site location: difference Fourier map
*R*[*F*^2^ > 2σ(*F*^2^)] = 0.034	Hydrogen site location: inferred from neighbouring sites
*wR*(*F*^2^) = 0.109	H-atom parameters constrained
*S* = 1.11	*w* = 1/[σ^2^(*F*_o_^2^) + (0.0527*P*)^2^ + 1.9073*P*] where *P* = (*F*_o_^2^ + 2*F*_c_^2^)/3
1775 reflections	(Δ/σ)_max_ < 0.001
128 parameters	Δρ_max_ = 0.23 e Å^−^^3^
0 restraints	Δρ_min_ = −0.23 e Å^−^^3^

### Special details

**Table:**

Geometry. Bond distances, angles *etc*. have been calculated using the rounded fractional coordinates. All su's are estimated from the variances of the (full) variance-covariance matrix. The cell e.s.d.'s are taken into account in the estimation of distances, angles and torsion angles
Refinement. Refinement of *F*^2^ against ALL reflections. The weighted *R*-factor *wR* and goodness of fit *S* are based on *F*^2^, conventional *R*-factors *R* are based on *F*, with *F* set to zero for negative *F*^2^. The threshold expression of *F*^2^ > σ(*F*^2^) is used only for calculating *R*-factors(gt) *etc*. and is not relevant to the choice of reflections for refinement. *R*-factors based on *F*^2^ are statistically about twice as large as those based on *F*, and *R*- factors based on ALL data will be even larger.

### Fractional atomic coordinates and isotropic or equivalent isotropic displacement parameters (Å^2^)

**Table:**

	*x*	*y*	*z*	*U*_iso_*/*U*_eq_	
Cl1	0.27943 (3)	0.9289 (2)	0.34233 (4)	0.0593 (3)	
S1	0.08011 (2)	0.46913 (17)	0.10724 (3)	0.0436 (2)	
O1	−0.03091 (7)	1.0496 (5)	0.16023 (11)	0.0610 (7)	
N1	0.06728 (7)	0.7792 (5)	0.21108 (10)	0.0383 (6)	
N2	−0.00930 (7)	0.7704 (5)	0.08375 (10)	0.0353 (6)	
C1	0.12315 (9)	0.7158 (6)	0.27507 (12)	0.0334 (7)	
C2	0.16843 (9)	0.8388 (6)	0.27522 (13)	0.0375 (8)	
C3	0.22127 (9)	0.7785 (6)	0.33950 (13)	0.0375 (7)	
C4	0.22942 (10)	0.6054 (7)	0.40248 (14)	0.0461 (8)	
C5	0.18373 (11)	0.4900 (7)	0.40138 (16)	0.0520 (9)	
C6	0.13025 (10)	0.5409 (6)	0.33725 (14)	0.0422 (8)	
C7	0.04606 (9)	0.6845 (5)	0.13794 (12)	0.0328 (7)	
C8	−0.04474 (9)	0.9441 (6)	0.09596 (14)	0.0395 (8)	
C9	−0.10196 (10)	0.9910 (7)	0.02487 (15)	0.0480 (9)	
H1	0.04591	0.88682	0.22087	0.0459*	
H2	−0.02292	0.70566	0.03650	0.0424*	
H3	0.16340	0.95861	0.23319	0.0450*	
H4	0.26537	0.56692	0.44526	0.0553*	
H5	0.18883	0.37642	0.44424	0.0624*	
H6	0.09947	0.45780	0.33622	0.0506*	
H9A	−0.09942	1.10785	−0.01390	0.0720*	
H9B	−0.12438	1.10907	0.03877	0.0720*	
H9C	−0.11887	0.78623	0.00402	0.0720*	

### Atomic displacement parameters (Å^2^)

**Table:**

	*U*^11^	*U*^22^	*U*^33^	*U*^12^	*U*^13^	*U*^23^
Cl1	0.0285 (4)	0.0836 (6)	0.0561 (5)	−0.0088 (3)	0.0164 (3)	0.0058 (4)
S1	0.0301 (4)	0.0600 (4)	0.0331 (4)	0.0091 (3)	0.0120 (3)	−0.0070 (3)
O1	0.0378 (11)	0.0981 (16)	0.0396 (11)	0.0147 (10)	0.0159 (9)	−0.0173 (10)
N1	0.0223 (10)	0.0590 (13)	0.0290 (10)	0.0031 (9)	0.0109 (8)	−0.0061 (9)
N2	0.0231 (9)	0.0509 (12)	0.0251 (10)	0.0023 (8)	0.0086 (8)	−0.0049 (8)
C1	0.0248 (11)	0.0434 (13)	0.0262 (12)	0.0014 (9)	0.0099 (9)	−0.0051 (9)
C2	0.0303 (13)	0.0479 (14)	0.0283 (12)	−0.0001 (10)	0.0119 (10)	0.0032 (10)
C3	0.0250 (12)	0.0480 (14)	0.0334 (12)	−0.0038 (10)	0.0117 (10)	−0.0051 (10)
C4	0.0302 (13)	0.0605 (16)	0.0342 (14)	0.0060 (11)	0.0085 (11)	0.0070 (12)
C5	0.0439 (16)	0.0684 (18)	0.0374 (14)	0.0033 (13)	0.0178 (13)	0.0173 (13)
C6	0.0340 (14)	0.0545 (15)	0.0383 (14)	−0.0041 (11)	0.0196 (11)	0.0004 (11)
C7	0.0242 (12)	0.0400 (13)	0.0298 (12)	−0.0014 (9)	0.0116 (10)	−0.0003 (10)
C8	0.0274 (13)	0.0504 (15)	0.0371 (14)	0.0019 (10)	0.0150 (11)	−0.0017 (11)
C9	0.0304 (14)	0.0653 (17)	0.0412 (14)	0.0124 (12)	0.0146 (12)	−0.0002 (12)

### Geometric parameters (Å, º)

**Table:**

Cl1—C3	1.739 (3)	C3—C4	1.373 (4)
S1—C7	1.667 (3)	C4—C5	1.373 (5)
O1—C8	1.222 (3)	C5—C6	1.382 (4)
N1—C1	1.431 (3)	C8—C9	1.493 (4)
N1—C7	1.324 (3)	C2—H3	0.9300
N2—C7	1.394 (3)	C4—H4	0.9300
N2—C8	1.367 (4)	C5—H5	0.9300
N1—H1	0.8600	C6—H6	0.9300
N2—H2	0.8600	C9—H9A	0.9600
C1—C6	1.374 (3)	C9—H9B	0.9600
C1—C2	1.383 (4)	C9—H9C	0.9600
C2—C3	1.379 (4)		
			
C1—N1—C7	124.9 (2)	N1—C7—N2	116.3 (2)
C7—N2—C8	128.42 (19)	O1—C8—C9	122.5 (3)
C1—N1—H1	118.00	N2—C8—C9	114.8 (2)
C7—N1—H1	118.00	O1—C8—N2	122.7 (2)
C7—N2—H2	116.00	C1—C2—H3	121.00
C8—N2—H2	116.00	C3—C2—H3	121.00
N1—C1—C2	120.5 (2)	C3—C4—H4	120.00
N1—C1—C6	118.1 (3)	C5—C4—H4	120.00
C2—C1—C6	121.3 (2)	C4—C5—H5	120.00
C1—C2—C3	118.1 (2)	C6—C5—H5	120.00
Cl1—C3—C4	118.6 (2)	C1—C6—H6	120.00
C2—C3—C4	121.7 (3)	C5—C6—H6	120.00
Cl1—C3—C2	119.68 (19)	C8—C9—H9A	109.00
C3—C4—C5	119.1 (3)	C8—C9—H9B	109.00
C4—C5—C6	120.7 (3)	C8—C9—H9C	109.00
C1—C6—C5	119.2 (3)	H9A—C9—H9B	109.00
S1—C7—N1	125.0 (2)	H9A—C9—H9C	109.00
S1—C7—N2	118.65 (16)	H9B—C9—H9C	109.00
			
C7—N1—C1—C2	63.5 (3)	C6—C1—C2—C3	0.6 (4)
C7—N1—C1—C6	−119.0 (3)	N1—C1—C6—C5	−176.9 (2)
C1—N1—C7—S1	1.1 (4)	C2—C1—C6—C5	0.6 (4)
C1—N1—C7—N2	−179.9 (2)	C1—C2—C3—Cl1	−179.9 (2)
C8—N2—C7—S1	179.9 (2)	C1—C2—C3—C4	−0.8 (4)
C8—N2—C7—N1	0.8 (4)	Cl1—C3—C4—C5	179.0 (2)
C7—N2—C8—O1	−0.3 (4)	C2—C3—C4—C5	−0.1 (4)
C7—N2—C8—C9	−179.5 (2)	C3—C4—C5—C6	1.3 (4)
N1—C1—C2—C3	178.0 (2)	C4—C5—C6—C1	−1.6 (4)

### Hydrogen-bond geometry (Å, º)

**Table:**

*D*—H···*A*	*D*—H	H···*A*	*D*···*A*	*D*—H···*A*
N1—H1···O1	0.86	1.96	2.648 (3)	136
N2—H2···S1^i^	0.86	2.56	3.4095 (18)	170
C9—H9*A*···S1^ii^	0.96	2.85	3.799 (3)	170

Symmetry codes: (i) −*x*, −*y*+1, −*z*; (ii) −*x*, −*y*+2, −*z*.
